# Simultaneous determination of 14 analgesics in postoperative analgesic solution by HPLC–DAD and LC–MS/MS

**DOI:** 10.1186/s13065-024-01113-6

**Published:** 2024-01-10

**Authors:** Manman Yao, Baoxia Fang, Jinguo Yang, Sicen Wang, Fuchao Chen

**Affiliations:** 1 Sinopharm Dongfeng General Hospital, Hubei University of Medicine, Shiyan, Hubei 442008 People’s Republic of China; 2 School of Pharmacy, Hubei University of Medicine, Shiyan, Hubei 442000 People’s Republic of China; 3 School of Pharmacy, Xi’an Jiaotong University, Xi’an, 710061 Shanxi People’s Republic of China

**Keywords:** HPLC–DAD, LC–MS/MS, Opioids, Postoperative pain, Separate

## Abstract

A green, efficient, sensitive and accurate detection method by HPLC–DAD and LC–MS/MS was developed and validated for the quantification of morphine, hydromorphone, oxycodone, ketamine tramadol, dezocine, ropivacaine, remifentanil, butorphanol, bupivacaine, droperidol, fentanyl, lornoxicam and sufentanil. The 14 mixtures were chromatographed via HPLC–DAD method which employed 0.05 mol/L potassium dihydrogen phosphate solution-acetonitrile as the mobile phase, the analytes were gradient elution on a SinoChrom ODS-BP C_18_ column with a total separation time of 35 min, and 14 mixtures showed a good linear relationship in the linear range. The Limit of Quantitation (LOQ) ranged from 0.10 to 20.0 µg/mL, the inter-day and intra-day precision of each analyte is within 1.1–2.0% and 0.4–1.3%, and the average absolute recovery of all compounds was above 98%. The LC–MS/MS method was used to successfully separate the 14 mixtures within 10 min which employed 0.1% formic acid-acetonitrile as the mobile phase, the analytes were gradient elution on a ACQUITY UPLC-BEH C_18_ column with a total separation time of 13 min, and 14 mixtures showed a good linear relationship in the linear range. The LOQ ranged from 0.005 to 0.2 ng/mL, the inter-day and intra-day precision of each analyte is within 1.2–4.1% and 0.6–3.3%, and the average absolute recovery of all compounds was above 93%. The proposed method has been successfully applied in the clinic and provides a strong technical basis for the quantitative detection of these 14 mixtures for detecting drug abuse, and for studying the stability and compatibility of analgesic solutions. The proposed methods were validated against ICH guidelines.

**Supplementary Information:**

The online version contains supplementary material available at 10.1186/s13065-024-01113-6.

## Introduction

Postoperative pain is a reaction after tissue injury, which not only affects the recovery of postoperative patients, but also brings pain and burden to patients and their families [1]. In 1995, the American Pain Society listed pain as the “fifth vital sign”, raising awareness of the need for pain management. Postoperative pain can be divided into acute pain and chronic pain. Studies have found that the medical costs of acute and chronic pain in the United States exceed those of cancer, diabetes, and heart disease combined [2, 3], and its treatment options create much more difficulties than expected, so an effective postoperative analgesic treatment plan is extremely important.

The treatments for postoperative pain include drug therapy and physical therapy, of which drug therapy is the main method. Commonly used postoperative analgesics include opioid analgesics, nonsteroidal anti-inflammatory drugs, local anesthetics, auxiliary analgesics, etc. [4]. Among these, opioids are the first choice for patients with acute or chronic pain. In the 1980s and 1990s, the decline of multidisciplinary pain treatment clinics and the mass marketing of new oxycodone extend-release products led to the increasing use of opioids by medical institutions and medical personnel for the relief of chronic noncancerous pain [5]. In this environment, more patients choose opioids for pain relief. In theory, opioids have no capping effect on analgesia, and no matter how strong the pain is, increasing the dose can achieve effective control, but higher doses can lead to serious adverse reactions and addiction [6]. In recent years, opioids (natural or modified compounds of poppy plants) and novel synthetic opioids (synthetic chemicals that act on opioid receptors) have played an irreplaceable role in the field of postoperative pain treatment, with the U.S. Food and Drug Administration, the Centers for Disease Prevention and Control (CDC), and several medical organizations finding that opioid use is increasing, that opioid addiction and mortality rates are also on the rise [7–9], and that prescription opioids may be the most important contributor to drug addiction and dependence [10–12]. Despite evidence that the increase in opioid prescriptions leads to opioid abuse, opioid prescribing remains a common and increasing practice in hospital settings, especially for the treatment of chronic pain [13]. The monitoring of opioid abuse in China is still in the early stages, while the problem is not as severe as in the United States, how to prevent opioid abuse when ensuring proper pain treatment is a challenging and urgent issue.

In recent decades, the concepts of multimodal analgesia (MMA) and patient-controlled analgesia (PCA) have been strongly advocated by domestic and foreign scholars. PCA is a kind of analgesic parameter preset by medical staff according to the patient's age, weight, type of surgery and physical condition, such as load dose, background dose, self-control dose and locking time, and PCA is a common “self-management” pain treatment technique for patients after surgery [14, 15]. Although the analgesics of this method are effective in relieving pain, they can produce some serious side effects, including respiratory depression, nausea, vomiting, dizziness, itching, and confusion [16–18]. Multimodal analgesia (MMA) is a good alternative to PCA. The concept of MMA was introduced more than 20 years ago as a technique to improve analgesia and reduce the incidence of opioid-related adverse events [19]. It uses combinations of analgesic drugs with different mechanisms of action to achieve pain control, reduce opioid consumption, and reduce drug-related side effects [20]. As reported in many studies, MMA regimens vary from patient to patient [21, 22]. In addition, domestic and foreign medical institutions lack commercial analgesic liquid preparations that can be given directly to the patient. Usually, two or more analgesic drugs are put into a disposable analgesic pump in the department of anesthesiology for patients to use for 4–48 h, though some patients can use it for 7–15 days [23, 24]. How to avoid mistakes in the formulation of analgesic solutions, prevent the abuse of illicit drugs, ensure the quality of the formulations, and ensure the compatibility and stability of analgesic solutions, have been challenges in popularizing MMA.

**Fig. 1:**
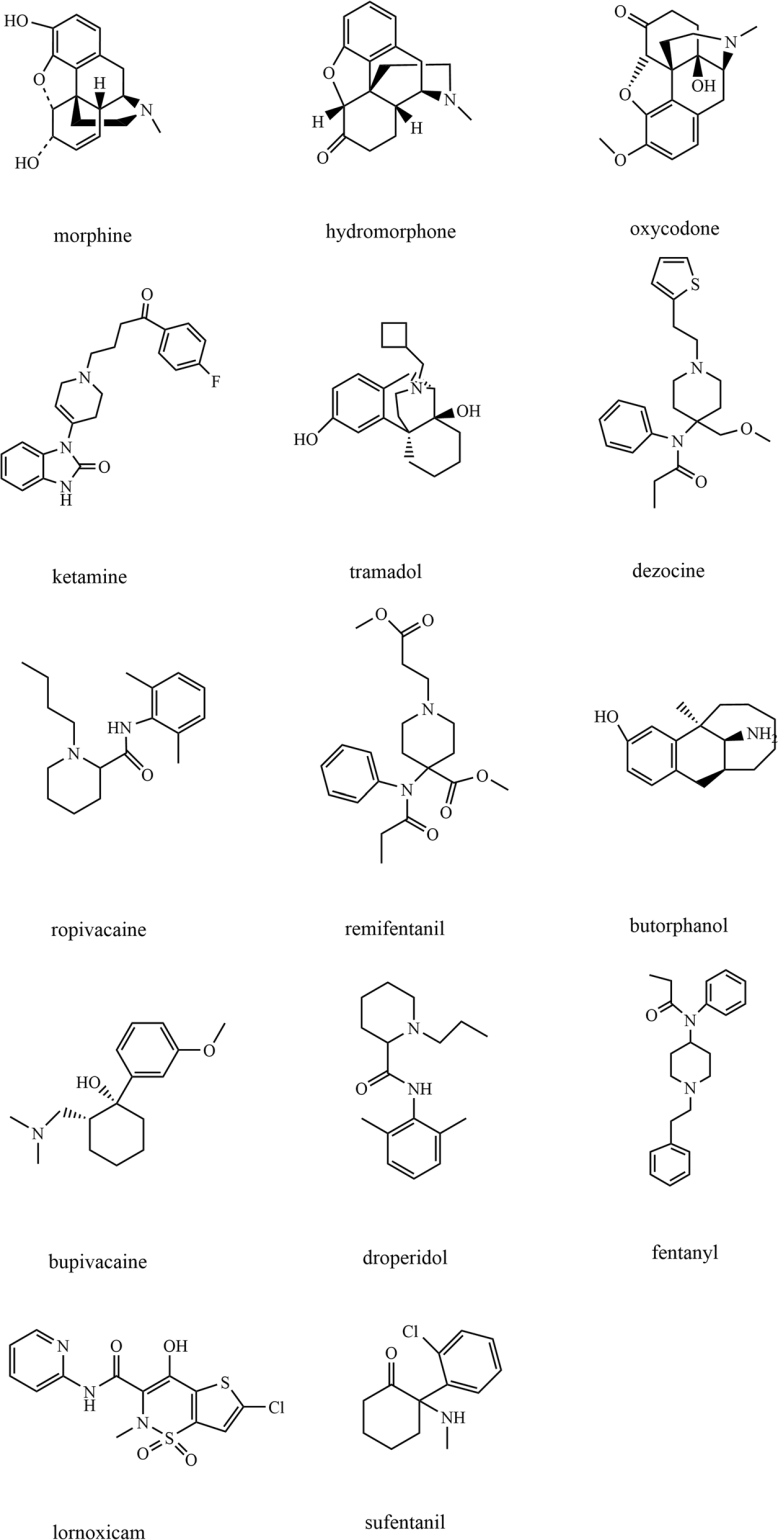
The chemical structure of 14 analgesics

Based on the above problems, we established an HPLC–DAD and LC–MS/MS method for simultaneous measurement of 14 analgesics in postoperative MMA-PCA analgesic solution, namely, morphine, hydromorphone, oxycodone, ketamine tramadol, dezocine, ropivacaine, remifentanil, butorphanol, bupivacaine, droperidol, fentanyl, lornoxicam and sufentanil, (Fig. 1). HPLC–DAD method is the use of liquid chromatography column to separate the compounds in the mixture, and then the Diode Array Detector for qualitative and quantitative analysis of each compound. LC–MS/MS is a combination of liquid chromatography and mass spectrometry, liquid chromatography is responsible for separating the object to be measured from the interference, and mass spectrometry is responsible for detection and qualitative and quantitative analysis of the object to be measured. We also used this method for quality monitoring and stability analysis of postoperative multimode analgesic solution, which can guarantee the safe and reasonable use of postoperative analgesic drugs.

## Methods

### Materials and reagents

Morphine hydrochloride (purity 87.3%), oxycodone hydrochloride (purity 94.9%), ketamine hydrochloride (purity 99.7%), tramadol hydrochloride (purity 99.8%), dezocine (purity 100.0%), ropivacaine hydrochloride (purity 94.5%), remifentanil hydrochloride (purity 100.0%), butorphanol tartrate (purity 99.7%), bupivacaine hydrochloride (purity 95.0%), droperidol (purity 97.8%), fentanyl citrate (purity 99.9%), lornoxicam (purity 99.6%), and sufentanil citrate (purity 99.4%) control products were purchased from China National Institute of Food and Drug Control (Beijing, China) and hydromorphone hydrochloride (purity 99.0%) were supplied from Yichang Renfu Pharmaceutical Co., Ltd. Morphine hydrochloride injection (1 mL: 10 mg) (Shenyang First Pharmaceutical Co., Ltd.), hydromorphone hydrochloride injection (2 mL: 2 mg) (Yichang Renfu Pharmaceutical Co., Ltd.), oxycodone hydrochloride injection (2 mL: 10 mg) (Mengdi China Pharmaceutical Co., Ltd.), ketamine hydrochloride injection (2 mL: 100 mg) (Jiangsu Hengrui Pharmaceutical Co., Ltd.), tramadol hydrochloride injection (2 mL: 100 mg) (Duoduo Pharmaceutical Co., Ltd.), dezocine injection (1 mL: 5 mg) (Yangzijiang Pharmaceutical Group Co., Ltd.), ropivacaine hydrochloride injection (10 mL: 10 mg) (Yichang Renfu Pharmaceutical Co., Ltd.), remifentanil for injection (1 mg) (Yichang Renfu Pharmaceutical Co., Ltd.), butorphanol tartrate injection (2 mL: 4 mg) (Jiangsu Hengrui Pharmaceutical Co., Ltd.), bupivacaine hydrochloride injection (5 mL: 25 mg) (Shanghai Hefengye Pharmaceutical Co., Ltd.), droperidol injection (2 mL: 5 mg) (Shanghai Xudong Haipu Pharmaceutical Co., Ltd.), fentanyl Citrate injection (2 mL: 0.1 mg) (Yichang Renfu Pharmaceutical Co., Ltd.), lornoxicam injection (8 mg) (Zhejiang Zhenyuan Pharmaceutical Co., Ltd.), sufentanil citrate injection (2 mL: 100 μg) (Yichang Renfu Pharmaceutical Co., Ltd.). All of the above medicines were purchased from Sinopharm Holding Company Limited (Hubei, China). HPLC-grade acetonitrile and formic acid were purchased from Tianjin Kemiou Chemical Reagent Co., Ltd. (Tianjing, China), potassium dihydrogen phosphate was purchased from Shanghai Pudong Chemical Reagent Factory (Shanghai, China), and 0.9% sodium chloride injection was purchased from Wuhan Binhu Shuanghe Pharmaceutical Co., Ltd (Wuhan, China).

### Instrumentation

Instruments were the DIONEX Ultimate 3000 High performance Liquid Chromatograph (Dionex, Germany), including the Ultimate 3000 four-element low-pressure gradient pump, the Ultimate 3000 diode array detector, and Chromeleon chromatographic workstation; the AB SCIEX QTRAP 6500 + Liquid Mass Spectrometer (SCIEX), consisting of an ultrahigh-performance liquid chromatography (UPLC) instrument and triple-quadrupole linear ion trap mass spectrometry (QTRAP) instrument, equipped with an ExionLC AD pump, ExionLC AD automatic sampler, LTSC-0873 column temperature box and ExionLC controller; and a data analysis R1.7.2. MS105DU electronic balance (Mettler Toledo, Switzerland).

### Chromatographic conditions

#### HPLC–DAD determination

A SinoChrom ODS-BPC_18_ (5 μm, 150 mm × 4.6 mm) was used for chromatographic separation. The mobile phase comprised A (0.05 mol/L potassium dihydrogen phosphate aqueous solution) and B (acetonitrile), the analytes were gradient elution (Additional file 1: Table S1). The flow rate was 1.0 mL/min. The temperature of the column chamber was 30 °C, and the injection volume was 20 μL. The detection wavelength was 280 nm for morphine, hydromorphone, oxycodone, dezocine, butorphanol, droperidol and lornoxicam; 270 nm for ketamine and tramadol; 263 nm for ropivacaine and bupivacaine; and 215 nm for fentanyl, sufentanil and remifentanil.

#### LC–MS/MS determination

A Waters ACQUITY UPLC^®^ BEH C_18_ column (100 mm × 2.1 mm, 1.7 µm) was used for chromatographic separation. The mobile phase comprised solutions A (acetonitrile) and B (0.1% formic acid aqueous solution), the analytes were gradient elution (Additional file 1: Table S2). The flow rate was 0.5 mL/min. The temperature of the column chamber was 40 °C, and the injection volume was 1 μL. ESI was set to positive ionization mode. The mass spectrum parameters were as follows: capillary voltage, 4000 V; spray gas pressure, 344.75 kPa; dry gas flow rate, 10 L/min; curtain gas (CUR), 35 psi; collision gas (CAD), 9 psi; ion spray voltage (IS), 5500 V (ESI); temperature (TEM), 550 °C; ion source gas 1 (GS1), 55 psi; and ion source gas 2 (GS2), 55 psi. The scanning time was 0.98 ms, with a total time of 13 min. The mass spectrum parameters of all 14 analgesic drugs are shown in Table 1.

**Table 1:** Q1/Q3 masses and compound-specific parameters for both MRM transitions of each analyte

Analyte name w/MRM #	Retention time (t_R_)	Molecular formula	Elative molecular mass	Q1 Mass	Q3 Mass	DP	CE
Morphine	1.02	C_17_H_20_CLNO_3_	285.30	286.1	153.1	100	53
					201.0*	100	36
Hydromorphone	1.44	C_17_H_19_NO_3_	285.34	286.0	199.1	100	40
					185.0*	100	40
Oxycodone	2.43	C_l8_H_21_NO_4_	315.33	316.2	256.1	80	36
					241.1*	80	36
Ketamine	3.44	C_13_H_17_CL_2_NO	237.69	238.1	179.0	28	18
					125.0*	28	25
Tramadol	4.57	C_16_H_26_CLNO_2_	263.34	264.2	159.0	50	37
					58.0*	50	37
Dezocine	4.9	C_16_H_23_NO	245.36	246.2	147.1	80	28
					229.1*	80	14
Ropivacaine	5.66	C_17_H_26_N_2_O	274.36	275.3	84.0	100	40
					126.2*	100	40
Remifentanil	6.97	C_20_H_28_N_2_O_5_	376.45	377.2	113.1	65	38
					317.2*	65	22
Butorphanol	7.17	C_21_H_29_NO_2_	327.46	328.0	282.0	100	40
					242.0*	100	40
Bupivacaine	7.37	C_18_H_28_N_2_O	288.39	289.3	84.1	40	51
					140.2*	40	29
Droperidol	7.59	C_22_H_22_FN_3_O_2_	379.43	380.1	194.0	100	40
					165.0*	100	47
Fentanyl	7.87	C_22_H_28_N_2_O	336.47	337.0	105.0	70	31
					188.0*	70	25
Lornoxicam	9.29	C_13_H_10_CLN_3_O_4_S_2_	371.82	372.0	164.0	100	40
					121.0*	100	40
Sufentanil	9.61	C_22_H_30_N_2_O_2_S	386.55	387.3	111.2	50	55
					238.1*	50	27

### Preparation of the stock and standard curve solutions

Control products of morphine hydrochloride, oxycodone hydrochloride, hydromorphone hydrochloride, fentanyl citrate, sufentanil citrate, remifentanil hydrochloride, dezocine, butorphanol tartrate, tramadol hydrochloride, bupivacaine hydrochloride, ropivacaine hydrochloride, droperidol, ketamine hydrochloride and lornoxicam were taken in appropriate amounts and precision weighed, which was placed in 25 mL volumetric bottles and fixed with 0.9% sodium chloride solution. The standard reserve liquid of the prepared drug with concentrations of 0.8 mg/mL, 0.8 mg/mL, 0.8 mg/mL, 0.5 mg/mL, 0.5 mg/mL, 0.5 mg/mL, 0.9 mg/mL, 1.0 mg/mL, 0.8 mg/mL, 2.0 mg/mL, 2.0 mg/mL, 0.5 mg/mL, 1.0 mg/mL and 0.8 mg/mL was e stored at − 20℃ prior to use.

### Method validation

The linear range, Limit of Quantitation (LOQ), Limit of Detection (LOD), precision, repeatability, stability, recovery and other parameters of all 14 mixtures were verified according to the 2020 edition of the Chinese Pharmacopoeia and ICH analytical method validation guidelines.

#### linearity and LOQ

Calibration curves of standards in HPLC–DAD method were drawn by the concentration of the analyte (X) and the peak area of the chromatographic peak of the analyte (Y). Calibration curves of standards in LC–MS/MS method were drawn by the concentration of the analyte (X) and the peak area of MRM ion chromatographic peak of the analyte (Y). The correlation coefficient (*r*^2^) of the calibration curves was set to ≥ 0.990. LOD of 14 mixtures under the condition of a signal-to-noise ratio (S/N) should be no less than 3 and the LOQ of 14 mixtures under the condition of S/N should be no less than 10.

#### Repeatability and precision

The reproducibility test was performed by taking appropriate amounts of the above 14 control products, diluting them in 0.9% sodium chloride solution, preparing 6 dilutions in parallel, measuring them, recording their peak areas, and calculating the RSD of each drug concentration. The precision was determined by repeated injection of 3 different concentrations of reference mixture on the same day 6 times, and the results from 3 straight days were used to determine the inter- and intra-day precisions (RSD).

#### Stability

In the sample stability test, the prepared sample solution was injected at different times, the peak area was recorded, the drug content was calculated, and the stability time was determined according to the RSD value.

#### Recovery

The recovery rate test was done by adding the control product solution to the known sample, injecting the sample, recording the peak area, calculating the drug content, subtracting the measured amount of the drug content before adding the sample and then dividing by the control product amount added to obtain the recovery rate of the sample.

### Quality monitoring of drug infusion preparation

#### Collection of infusion samples in the clinical ward

The whole study protocol was approved (LW-2023–039) by the ethical committee of Sinopharm Dongfeng General Hospital, Hubei University of Medicine. All participants gave their written informed consent. This work and all the methods described in this study were performed in accordance with our local institutional guidelines and regulations.

(1) Collection of sufentanil infusion samples.

ICU (intensive care unit) sampling: From May to June 2023, sufentanil postoperative analgesic solution prepared by nursing staff was collected at 8:30–9:30 AM and 2:30–3:00 PM. The concentration of sufentanil samples was 2 µg/mL (for postoperative intravenous injection). Each sample was drawn up with a 1 mL disposable syringe and placed in a 1.5 mL EP tube. Label records were made, and the samples were refrigerated at − 20 °C until testing.

(2) Collection of remifentanil infusion samples.

Anesthesiology sampling: From May to June 2023, a special person was asked to extract the postoperative analgesic solution of remifentanil in the operating room every day. The concentration of remifentanil was 20 µg/mL (for intravenous injection during surgery). Each sample was sampled with a 1 mL disposable syringe and placed in a 1.5 mL EP tube. Label records were made, and the samples were refrigerated at − 20 °C until testing.

(3) Collection of transfusion samples for lornoxicam.

Ward sampling after PIVAS (pharmacy intravenous admixture services) deployment: From May to June 2023, the postoperative analgesia solution prepared by PIVAS was collected in our oncology and pain departments from 8:30–9:30 AM. The sample concentration of lornoxicam was 16 mg/100 mL, and 1 mL was taken with a 1 mL disposable syringe for each sample, placed in a 1.5 mL EP tube, labeled, and refrigerated at − 20 °C until testing.

#### Determination of drug content in infusion samples

Sufentanil postoperative analgesia solution in the ICU, remifentanil postoperative analgesia solution in the anesthesiology department and lornoxicam postoperative analgesia solution in the PIVAS after blending were thawed at room temperature and then mixed well after 1 min. Then, 100 µL of the sample solution of lornoxicam was placed into a 1.5 mL EP tube, 900 mL of sodium chloride solution was added, and the tube was mixed and injected into the sample. Sufentanil and remifentanil samples were directly injected and analyzed according to the chromatographic conditions in Sect. "Chromatographic conditions". The peak area was recorded and substituted into the regression equation to calculate the sample concentration in the infusion sample. The relative percentage of each drug labeled dose was calculated based on the clinical administration concentration.

#### Statistical analysis of data

The relative percentages of sufentanil, remifentanil and lornoxicam samples were recorded in Excel tables, and the qualified rate of the three kinds of drug infusion was calculated with the drug content equivalent to 90%-110% of the labeled content as the qualification standard. Scatter plots of the content distribution of each sample were drawn by GraphPad Prism, and the quality of postoperative analgesic drug allocation in the ICU, anesthesiology department and PIVAS was investigated.

### Assessment of the proposed method’s greenness

Analytical GREEnness metric (AGREE) conducted a green evaluation of the established method. AGREE is calculated based on the 12 basic principles of the GAC. The center of the circular hieroglyphics is the final score, which is a score of one unit, from 0 to 1. The tool is accessed via the link mentioned in the AGREE publication [25].

## Results

### System adaptability test results

**Fig. 2:**
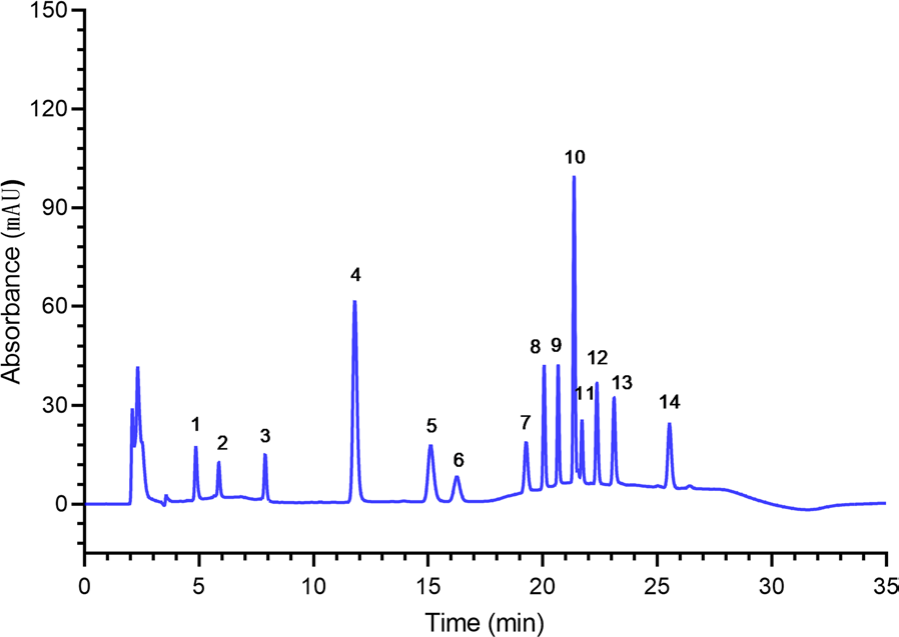
Chromatogram of the standard drug mixture sample obtained after 20 µL injection (HPLC–DAD). Notes: 1. morphine, 2. hydromorphone, 3. oxycodone, 4. ketamine, 5. tramadol, 6. dezocine, 7. ropivacaine, 8. remifentanil, 9. butorphanol, 10. bupivacaine, 11. droperidol, l2. fentanyl, 13. lornoxicam, 14. sufentanil

The HPLC–DAD method was used to optimize the composition of the mobile phase and the gradient elution procedure. The chromatograms of the 14 analgesic and auxiliary analgesic drugs are shown in Fig. 2. The theoretical plate number of each chromatographic peak of the 14 drugs was greater than 3000, the impurity peak did not interfere with the determination of the principal component, the separation degree was greater than 1.5, and the separation was completed within 30 min (Table 2).

**Table 2:** System suitability parameters of the HPLC–DAD method

Analyte name	Retention time (t_R_)	Asymmetry factor (A)	Capacity factor (K)	Resolution factor (Rs)	Selectivity factor (α)	Number of theoretical plates (EP)	HETP (mm)
Morphine	4.85	1.39	0.94	5.47	1.43	10,655	0.0141
Hydromorphone	5.86	0.80	1.34	11.17	1.60	17,117	0.0088
Oxycodone	7.88	1.03	2.15	15.06	1.73	28,979	0.0052
Ketamine	11.79	1.20	3.72	8.31	1.36	19,782	0.0076
Tramadol	15.11	1.18	5.04	2.38	1.09	16,919	0.0089
Dezocine	16.25	1.09	5.50	7.89	1.22	17,245	0.0087
Ropivacaine	19.27	1.09	6.71	3.54	1.05	78,738	0.0019
Remifentanil	20.06	1.10	7.02	3.85	1.03	225,103	0.0007
Butorphanol	20.67	1.11	7.27	4.57	1.04	302,207	0.0005
Bupivacaine	21.37	1.07	7.55	2.29	1.02	304,259	0.0005
Droperidol	21.71	1.12	7.69	3.98	1.03	350,326	0.0004
Fentanyl	22.36	1.13	7.94	3.85	1.04	243,479	0.0006
Lornoxicam	23.11	1.17	8.24	9.48	1.12	194,541	0.0007
Sufentanil	25.53	1.15	9.21	2.64	1.04	114,393	0.0013

Reference values: ①A = 1 for a symmetric peak, ②K = 1–10, ③R_s_ > 2, ④α > 1, ⑤EP: Increase with the efficiency of the separation, ⑥HETP: The smaller the value, the higher the column efficiency

**Fig. 3:**
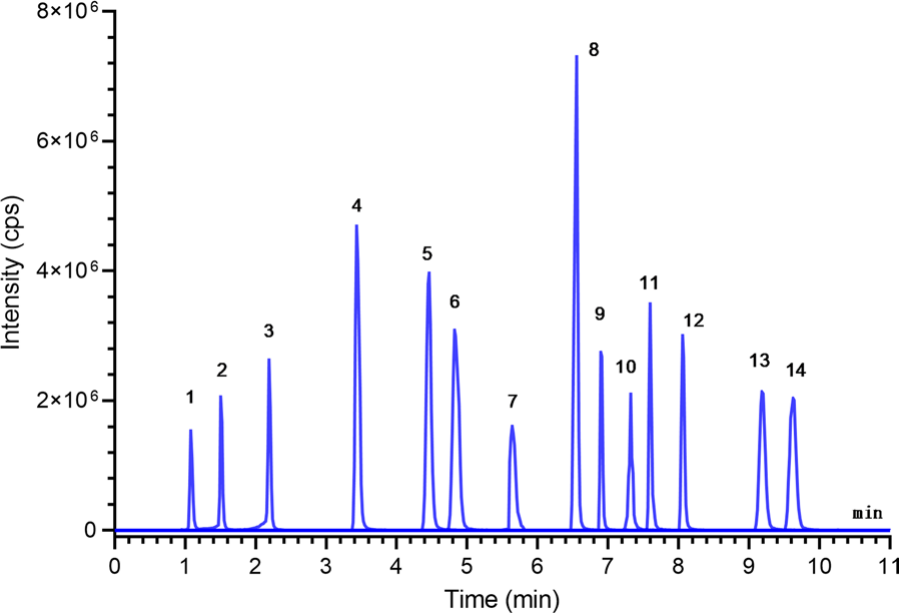
Chromatogram of the standard drug mixture sample obtained after 20 µL injection. (LC–MS/MS). Notes: 1. morphine, 2. hydromorphone, 3. oxycodone, 4. ketamine, 5. tramadol, 6. dezocine, 7. ropivacaine, 8. remifentanil, 9. butorphanol, 10. bupivacaine, 11. droperidol, l2. fentanyl, 13. lornoxicam, 14. sufentanil

The LC–MS/MS method was used to optimize the composition of the mobile phase and the gradient elution procedure. The chromatograms of all 14 analgesic and auxiliary analgesic drugs are shown in Fig. 3. The rapid separation and analysis of the 14 mixtures were realized by the LC–MS/MS method within 13 min. To achieve the highest signal-to-noise ratio and sensitivity of the ion flow chromatography of 14 mixtures as far as possible, after determining the parent ions of all 14 mixtures in this experiment, different CE ions of the parent ion fragment MS_2_ were collected and summarized, the optimal daughter ions were screened, and then the CE and DP voltages of each daughter ion were optimized by MRM. The ion mass spectrum fragments are shown in Figure S1 (Additional file 1).

### Methodological validation of HPLC–DAD and LC–MS/MS

The results of HPLC–DAD for the linear range and linear relationship, LOQ, LOD, precision, stability and recovery rate of the 14 analgesic and auxiliary analgesic drugs are shown in Table 3. As shown in the table, the RSD% of the inter- and intra-day precisions and drug concentration at different time points of the 14 drugs were below 2.0%, indicating good precision of the instrument. The prepared sample solution remained stable for 12 h at room temperature. The recoveries of analytes ranged from 98.2 to 101.9%, and the RSD was less than 2.0%, meaning that the recovery rate of the method was good.

**Table 3:** Assay validation parameters of the HPLC–DAD method

Analyte name	Linear equation	*r*	Linear Range (μg/mL)	LOD (μg/mL)	LOQ (μg/mL)	Stability (RSD%)	Recovery (RSD%)	Intra-day Precision (RSD%)	Inter-day Precision (RSD%)
Morphine	Y = 58.897X–2.3304	0.9999	20.0–200.0	0.15	0.50	0.9	99.6	0.9	1.5
Hydromorphone	Y = 7.7426X−2.1182	0.9996	20.0–200.0	0.20	0.60	1.4	99.4	0.4	1.1
Oxycodone	Y = 24.252X−3.9545	0.9998	20.0–200.0	0.30	1.00	0.8	98.2	0.9	1.6
Ketamine	Y = 6.4043X−2.0872	0.9991	20.0–200.0	2.50	8.00	1.7	100.2	0.6	1.1
Tramadol	Y = 9.2999X−10.616	0.9996	20.0–400.0	0.60	1.80	0.2	100.3	0.5	1.6
Dezocine	Y = 107.13X−23.168	0.9990	30.0–150.0	1.80	5.00	0.9	101.9	0.7	1.9
Ropivacaine	Y = 15.612X + 45.485	0.9998	50.0–500.0	7.50	20.00	1.6	99.2	1.2	1.9
Remifentanil	Y = 18.174X−0.3289	0.9996	0.5–10.0	0.07	0.20	1.2	99.4	1.3	1.6
Butorphanol	Y = 8.4406X + 13.256	0.9993	10.0–100.0	0.20	0.80	0.8	99.5	0.9	1.7
Bupivacaine	Y = 1.4093X + 7.1182	0.9992	50.0–500.0	2.00	6.00	1.0	101.1	1.3	2.0
Droperidol	Y = 9.9134X + 0.8142	0.9994	5.0–50.0	0.10	0.25	0.8	101.6	0.4	1.0
Fentanyl	Y = 70.895X−7.5161	0.9994	2.5–50.0	0.20	0.70	1.3	101.5	1.1	1.4
Lornoxicam	Y = 5.4772X−4.4635	0.9992	16.0–160.0	0.80	2.00	0.5	98.9	1.0	1.8
Sufentanil	Y = 63.32X−4.9491	0.9995	0.5–10.0	0.03	0.10	1.8	98.8	1.3	1.8

The results of the LC–MS/MS method for the linear range and linear relationship, LOD, LOQ, precision, stability and recovery rate of the 14 analgesic and auxiliary analgesic drugs are shown in Table 4. It can be seen from the table that the verification results of the method for all 14 drugs meet the requirements.

**Table 4:** Assay validation parameters of the LC–MS/MS method

Analyte name	Linear equation	*r*	Linear Range (μg/mL)	LOD (μg/mL)	LOQ (μg/mL)	Stability (RSD%)	Recovery (RSD%)	Intra-day Precision (RSD%)	Inter-day Precision (RSD%)
Morphine	Y = 15,123.4x−19903.9	0.9998	30.0–3000.0	0.030	0.100	0.6	100.7	3.3	4.1
Hydromorphone	Y = 146816x−24483.5	0.9991	3.6–360.0	0.040	0.110	1.1	97.4	2.7	3.5
Oxycodone	Y = 14,299.6x + 97,172.7	0.9987	4.25–425.0	0.040	0.130	1.2	95.4	1.4	2.5
Ketamine	Y = 16,904.4x + 219,548	0.9994	6.0–600.0	0.025	0.060	1.4	93.5	1.2	2.1
Tramadol	Y = 1039.3x + 7008.3	0.9995	3.0–300.0	0.060	0.200	0.4	100.1	1.0	1.2
Dezocine	Y = 3903.3x + 168,952	0.9992	30.0–3000.0	0.004	0.015	0.5	101.7	0.9	1.8
Ropivacaine	Y = 7110.7x + 7625.8	0.9996	1.0–100.0	0.002	0.005	1.8	98.3	1.3	2.5
Remifentanil	Y = 71,600.1x + 200,119	0.9998	1.5–150.0	0.002	0.005	1.3	95.4	1.6	3.9
Butorphanol	Y = 8804.7x + 134,721	0.9991	5.0–500	0.005	0.020	0.6	101.5	1.3	2.1
Bupivacaine	Y = 259878x + 93,774.5	0.9993	0.3–30.0	0.006	0.020	1.1	94.9	0.6	1.5
Droperidol	Y = 6196.8x + 34,185.8	0.9990	2.5–250.0	0.008	0.025	0.3	102.6	1.7	1.6
Fentanyl	Y = 61,017.9x + 90,943.7	0.9995	1.6–160.0	0.003	0.010	0.6	97.5	0.9	1.9
Lornoxicam	Y = 512.98x + 7110.4	0.9992	48.0–4800.0	0.048	0.192	1.0	98.8	1.4	2.7
Sufentanil	Y = 178346x + 133,993	0.9990	1.0–100.0	0.002	0.008	1.4	99.6	2.1	3.4

### Clinical infusion configuration quality monitoring results

**Fig. 4:**
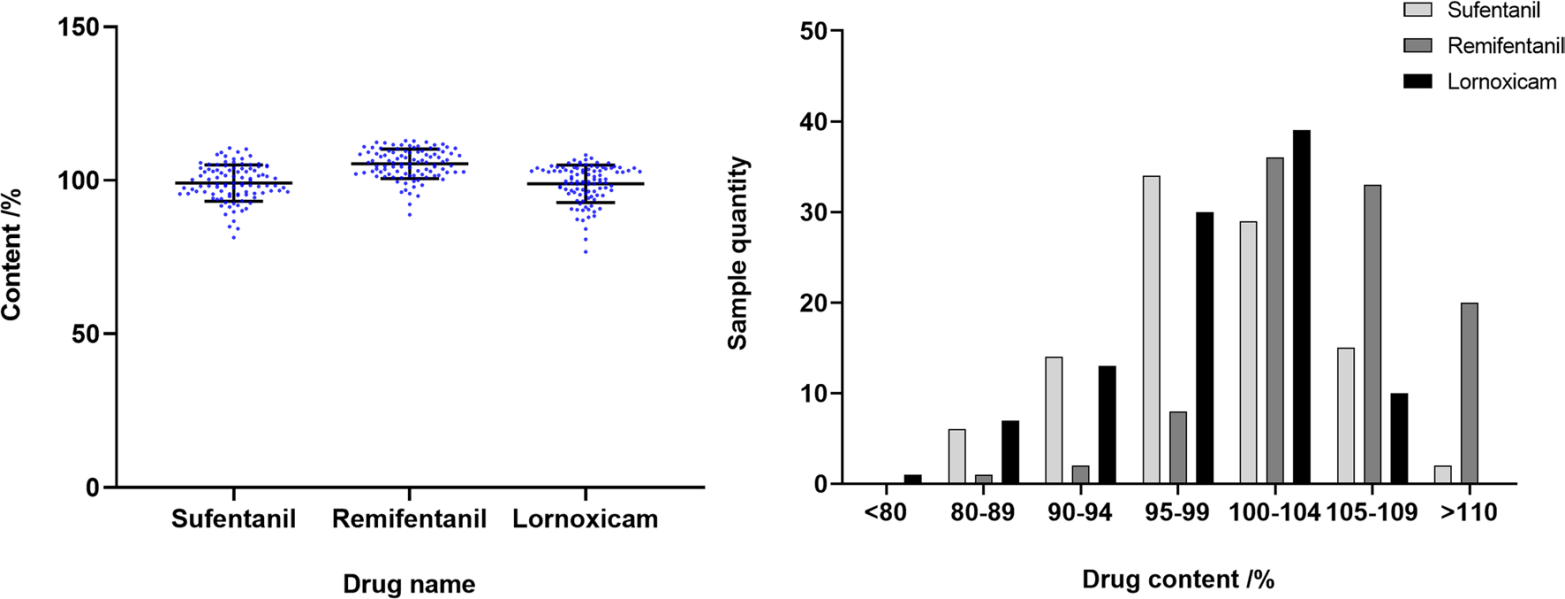
Clinical sample content monitoring results

One hundred postoperative analgesic fluids were taken from each of the two disease areas and PIVAS. The scatter diagram of the relative percentage content of the three postoperative analgesics and the distribution diagram of different content intervals are shown in Fig. 4. As seen from the figure, the acceptance rate of sufentanil in the ICU was 92%, that of remifentanil in anesthesiology was 79%, and that of lornoxicam in the PIVAS was 92%. Compared with the quality of infusion preparation in the anesthesiology department, the quality of infusion preparations in the ICU and PIVAS was better. Further work is needed to improve infusion configurations in hospitals.

### Assessment of the proposed method’s greenness

According to the AGREE evaluation tool, the score of HPLC–DAD is 0.74, and the score of LC–MS/MS is 0.71 (Additional file 1: Figure S2). The proposed HPLC–DAD and LC–MS/MS methods are more environmentally friendly, and the results show that the proposed technology has less impact on the environment.

## Discussion

In recent years, with the progress of surgical technology and the better understanding of postoperative analgesia, an effective postoperative analgesia program plays a pivotal role in alleviating the pain of patients, reducing the psychological and physiological stress reactions of patients in the perioperative period, reducing postoperative complications, promoting rehabilitation, and improving the postoperative quality of life.

Postoperative pain comes from the acute trauma and internal organ injury caused by the operation itself. Compared with single-mode analgesia, multimode analgesia is a more effective way to control postoperative pain by acting on different targets, reducing drug dosages and achieving more effective analgesia. Therefore, for postoperative patients, some clinical guidelines recommend the combination of two or more analgesics for PCIA to provide more effective analgesia. At present, opioid analgesics, nonsteroidal anti-inflammatory drugs, anesthesia, sedation and other drugs are widely used in combination for patients with self-controlled postoperative analgesia. This method can reduce the occurrence of adverse reactions while reducing the dose of a single drug and achieve a better analgesic effect [2, 26–29]. However, opioids and sedatives are strictly controlled drugs in China and elsewhere, and they come with a high risk of abuse. Therefore, it is of practical significance to establish an efficient, sensitive and economical detection method for the quality control, drug abuse prevention, serum or urine detection, compatibility, stability and pharmacokinetic study of analgesics.

From our review and analysis of domestic and foreign literature, there are many methods for the detection of single components of these drugs, including volume analysis, ultraviolet spectrophotometry, HPLC, GC and LC–MS. There are also a few reports on the simultaneous qualitative and quantitative analytical methods of various analgesics and auxiliary analgesics, but they are mainly limited to 2–6 drugs [30–43]. Wolf CE et al. [30] determined the contents of bupivacaine, clonidine, fentanyl, hydromorphone, midazolam and morphine in analgesic solution by HPLC, and applied this method to the quality detection of postoperative analgesic solution, finding the problem of illegal abuse of morphine by medical staff. Salmeron-GarciaA et al. [31] established HPLC method for quantitative analysis of tramadol, midazolam, bupivacaine and ropivacaine in analgesic solution, and used this method to investigate the compatibility stability of postoperative analgesic solutions of tramadol combined with three other drugs. Bodor GS [33] used LC–MS to quantify 43 chemical components commonly found in clinical urine, such as anesthetic analgesics, benzodiazepines and other abused drugs. Sorrieul J et al. [34] established a chromatographic method for simultaneous determination of intrathecal analgesics (morphine, ropivacaine, bupivacaine, baclofen, clonidine, sufentanil, fentanyl and ziconotide) according to the international ICH guidelines, and successfully applied it to quality control before the clinical use of PIVAS laminar flow table dispensing infusion. Jutras M [35] et al. adopted the LC–MS method with positive spray ionization and multireaction monitoring mode to qualitatively identify and quantitatively determine the principal components and metabolites of morphine hydromorphone, fentanyl, midazolam, and propofol in serum samples, and applied this method to the therapeutic drug monitoring of plasma samples from patients in intensive care units of several Canadian hospitals.

HPLC–DAD and LC–MS/MS methods for quantifying 14 postoperative analgesics were established in this paper. HPLC–DAD used 0.05 mol/L potassium dihydrogen phosphate aqueous solution-acetonitrile as the mobile phase for gradient elution, and the LC–MS/MS method used 0.1% formic acid-acetonitrile as the mobile phase in positive ion detection mode. The gradient elution procedure and the optimal chromatographic conditions for qualitative and quantitative analysis were determined by optimizing experiments. The above method is characterized by simple chromatographic conditions, fast analysis speed, high sensitivity and good accuracy, providing technical guidance for the quality evaluation of the above 14 postoperative analgesic drugs in hospitals, the quality evaluation of analgesic solution formulation, the detection of drug abuse, and the monitoring of the stability and compatibility of analgesic solutions.

## Conclusion

A green, efficient, sensitive and accurate chromatographic method was successfully developed and validated for the quantification of morphine, hydromorphone, oxycodone, ketamine tramadol, dezocine, ropivacaine, remifentanil, butorphanol, bupivacaine, droperidol, fentanyl, lornoxicam and sufentanil in clinically used analgesic mixture samples. Compared with the two methods, HPLC–DAD has the characteristics of low cost and high penetration rate, while LC–MS/MS has the characteristics of wide range, strong capability, reliable results, fast speed and detection limit, although the cost is high and the penetration rate is low. The two methods provide a strong technical guarantee for the quantification of these 14 postoperative analgesic drugs, for drug abuse detection, and for checking the stability and compatibility of analgesic solutions.

### Supplementary Information

**Additional file 1: Table S1.** Chromatography buffer gradient (HPLC-DAD). **Table S2.** Chromatography buffer gradient (LC-MS/MS). **Figure S1.** Mass spectrum of each analyte. Figure S2. AGREE score.

## Data Availability

The majority of the data used to support the findings of this study are included within the article. Other data are available from the corresponding author upon request.
