# Supplementary material for: Simultaneous determination of 14 analgesics in postoperative analgesic solution by HPLC–DAD and LC–MS/MS
Source: BMC Chem. 2024 Jan 10;18(1):10. doi: 10.1186/s13065-024-01113-6 (PMC10782708; doi:10.1186/s13065-024-01113-6)

**Supplementary Material**

**For**

**Simultaneous determination of 14 analgesics in postoperative analgesic**

**solution by HPLC-DAD and LC-MS/MS**

**Additional file 1: Table S1. Chromatography buffer gradient (HPLC-DAD)**

| Time after injection (min) | Buffer A (%) | Solvent B (%) |
| --- | --- | --- |
| 0 | 90 | 10 |
| 2 | 85 | 15 |
| 3 | 81 | 19 |
| 14 | 81 | 19 |
| 18 | 59 | 41 |
| 25 | 59 | 41 |
| 28 | 90 | 10 |
| 35 | 90 | 10 |

**Additional file 1: Table S2. Chromatography buffer gradient (LC-MS/MS)**

| Time after injection (min) | Solvent A (%) | Solvent B (%) |
| --- | --- | --- |
| 0 | 95 | 5 |
| 2 | 85 | 15 |
| 6 | 85 | 15 |
| 6.1 | 70 | 30 |
| 10 | 70 | 30 |
| 10.1 | 5 | 95 |
| 11 | 5 | 95 |
| 12.1 | 95 | 5 |
| 13 | 95 | 5 |

**Additional file 1: Figure S1. Mass spectrum of each analyte**

**
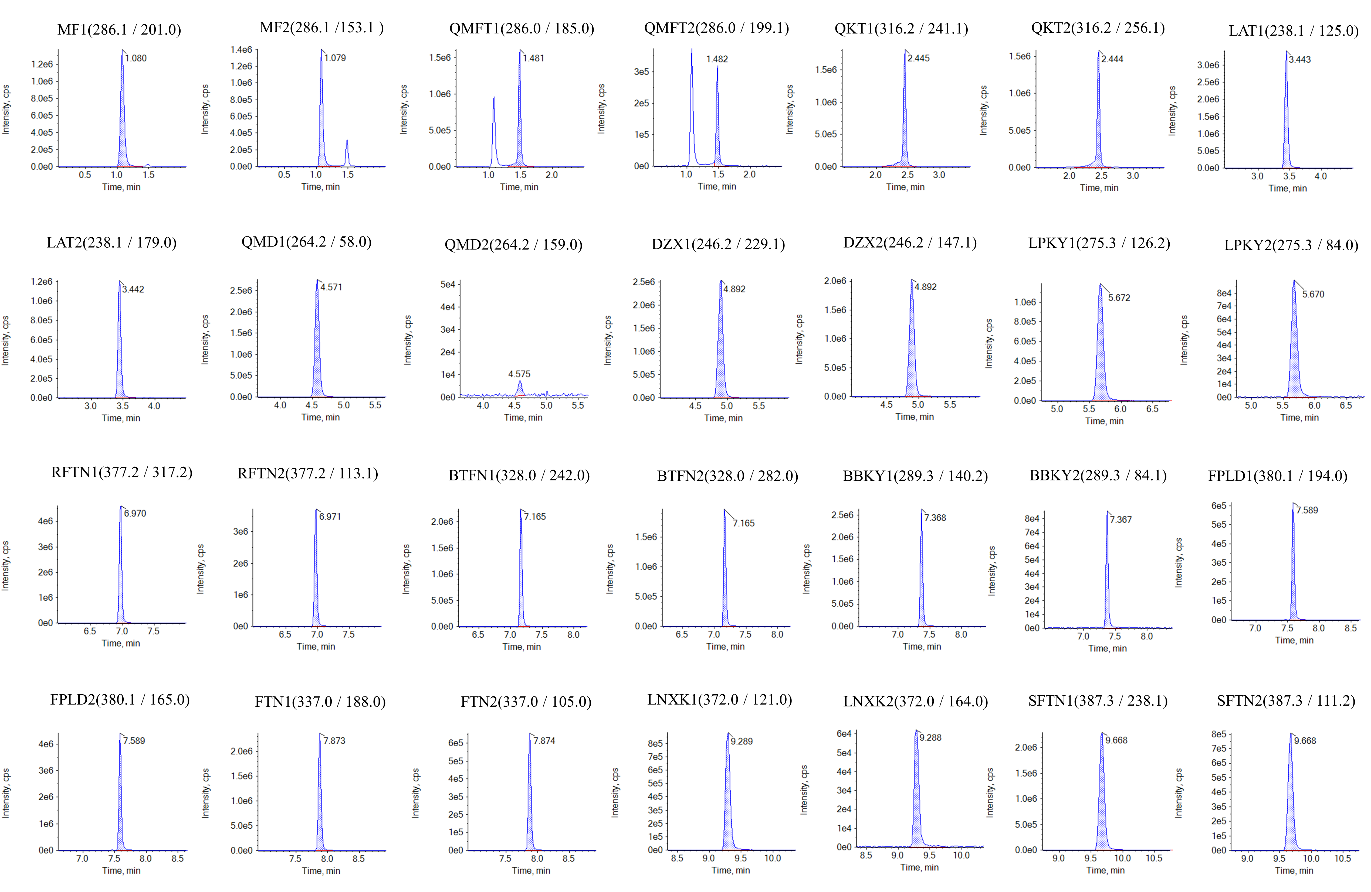
**

**Additional file 1: Figure S2. AGREE score**

Note: Score for 12 basic principles of HPLC-DAD: (1) 1.0 (2) 1.0 (3) 1.0 (4) 0.8 (5) 1.0 (6) 0.8 (7) 0.22 (8) 0.98 (9) 0.71 (10) 0.5 (11) 0.04 (12) 0.8

Score for 12 basic principles of LC-MS/MS: (1) 1.0 (2) 1.0 (3) 1.0 (4) 0.8 (5) 1.0 (6) 0.8 (7) 0.44 (8) 0.96 (9) 0.0 (10) 0.5 (11) 0.26 (12) 0.8


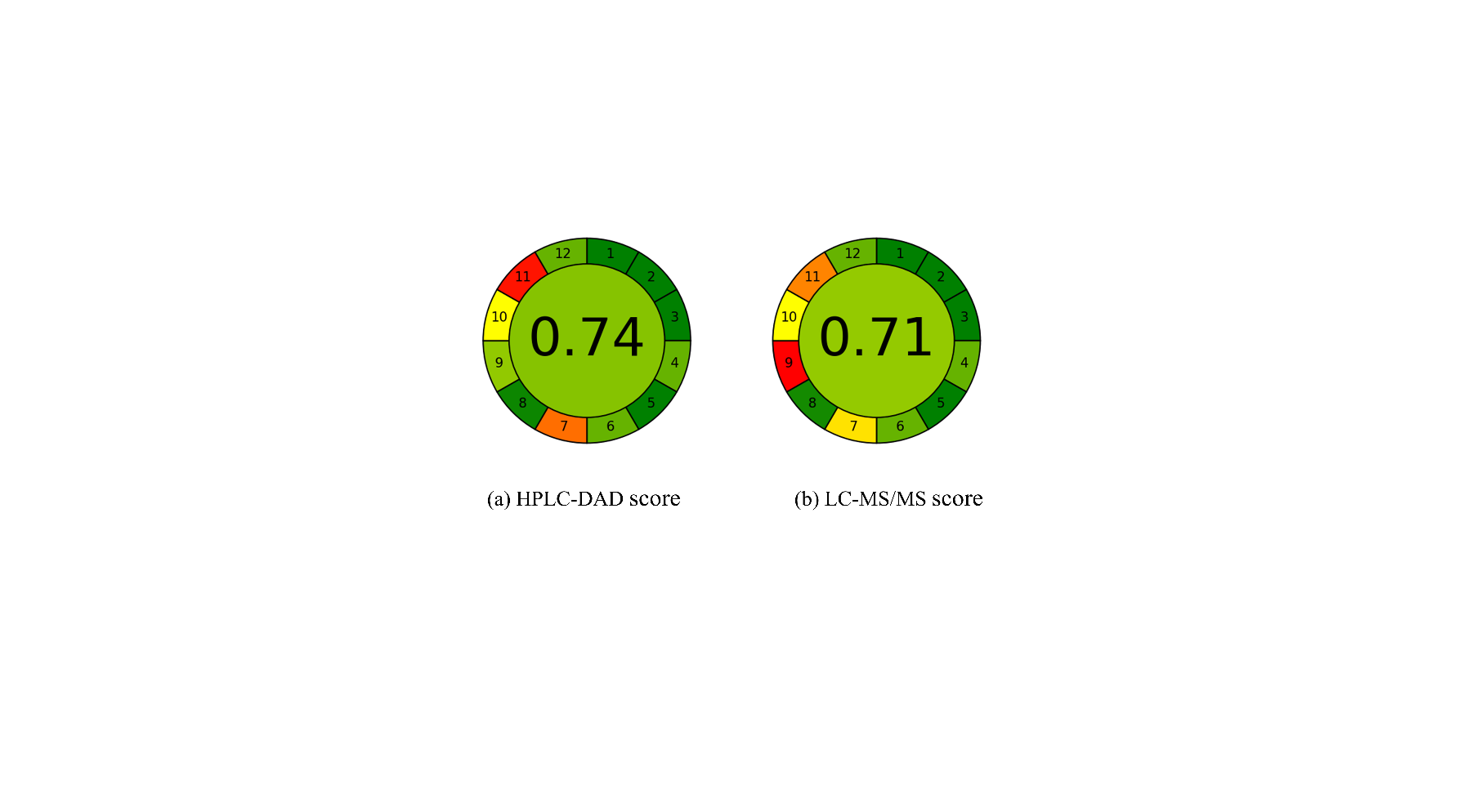

Supplement: Supplementary file 1 — Additional file 1: Table S1. Chromatography buffer gradient (HPLC-DAD). Table S2. Chromatography buffer gradient (LC-MS/MS). Figure S1. Mass spectrum of each analyte. Figure S2. AGREE score. [file 13065_2024_1113_MOESM1_ESM.docx]
